# Myocardial Noncompaction Presenting With Myocardial Bridge: A Case Report

**DOI:** 10.1097/MD.0000000000001425

**Published:** 2015-09-11

**Authors:** Yuechun Shen, Xinchun Li, Dongfeng Lu, Aiyi Xiao, Jun Li

**Affiliations:** From the Department of Cardiovascular Medicine (YS, DL); Department of Radiology (XL); Department of Ultrasound (AX); and Department of General Surgery (JL), The First Affiliated Hospital of Guangzhou Medical University, Guangzhou, China.

## Abstract

Myocardial noncompaction, namly isolated noncompaction of the left ventricular myocardium (NVM), is a rare congenital disease. It can be either seen in the absence of other cardiac anomalies, or associated with other congenital cardiac defects, mostly stenotic lesions of the left ventricular outflow tract. A myocardial bridge (MB) is thought being associated with coronary heart disease, such as coronary spasm, arrhythmia, and so on. The significance of MB in association with other congenital cardiac conditions is unknown.

We report a novel case who was presented NVM and MB. A 34-year-old man complained of chest prickling-like pain and dizzy for 1 year. His blood pressure was 110/70 mm Hg. Echocardiograph revealed increased trabeculations below the level of papillary muscle of left ventricle (LV); deep intertrabecular recesses in the endocardial wall of LV particularly in apex free wall; and LV ejection fraction of 57%. A coronary computerized tomography scan showed that part, 38.9 cm, of left descending artery tunnel was surrounding by cardiac muscles rather than resting on top of the myocardium.

The therapeutics interventions included lifestyle cares, agents of anti-ischemia and improvement myocardial cell metabolism. The patient was followed up for 2.6 years, and his general condition was stable.

This case indicates that NVM can be developed with MB, and the complete diagnosis of NVM and MB should be made by different image studies.

## INTRODUCTION

Myocardial noncompaction, namly isolated noncompaction of the left ventricular myocardium (NVM), is a rare congenital disorder caused by an arrest of compaction of the loose interwoven meshwork of myocardial fibers during embryogenesis. Clinical manifestations of it are highly variable, from no symptoms to arrhythmias, congestive heart failure, and systemic thromboemboli. It can be either seen in the absence of other cardiac anomalies, or associated with other congenital cardiac defects. A total of 12% of the patients with NVM have associated with other cardiac malformations, including various forms of congenital heart disease, particularly stenotic lesions of the left ventricular outflow tract (46%, mainly bicuspid aortic valve), Ebstein anomaly (25%), tetralogy of Fallot (8%), and double outlet right ventricle (4%).^1^

A myocardial bridge (MB) is defined as one of the coronary arteries tunnels through the myocardium rather than resting on top of it. MB was thought benign in the past. Currently, with the increasing studies, it is recognized clinically important mainly because of being associated with coronary heart disease, such as coronary spasm, arrhythmia, ischemia, acute coronary syndromes and sudden death.^2^ The significance of MB in association with other congenital cardiac conditions is unknown, although it was reported to accompany hypertrophic cardiomyopathy with significantly more frequent up to 41%.^3^

Both NVM and MB are congenital diseases. Currently, the diagnoses of the diseases sometimes are overlooked, missed, or delayed because of lack of the knowledge. Meanwhile, the diseases were discovered more chances than before as the new cardiac imaging technologies developed. Increasing the knowledge about the diseases is necessary. Here we report a parent who was presented both NVM and MB. Doctors should pay attention to the diseases, and beware that NVM can be developed with MB.

## CASE REPORT

A 34-year-old man, Han Chinese, self-employed garment worker, complained of chest prickling-like pain and dizzy for 1 year, visited our hospital in September 2012. He was ever diagnosed as low blood pressure and nervous breakdown. He was not smoking. His past medical history was unremarkable, but he was physically weak as he was young. He had no family history of heart diseases. His blood pressure was 110/70 mm Hg; heart rate was 72 beats/min. Physical examination was not remarkable.

**FIGURE 1:**
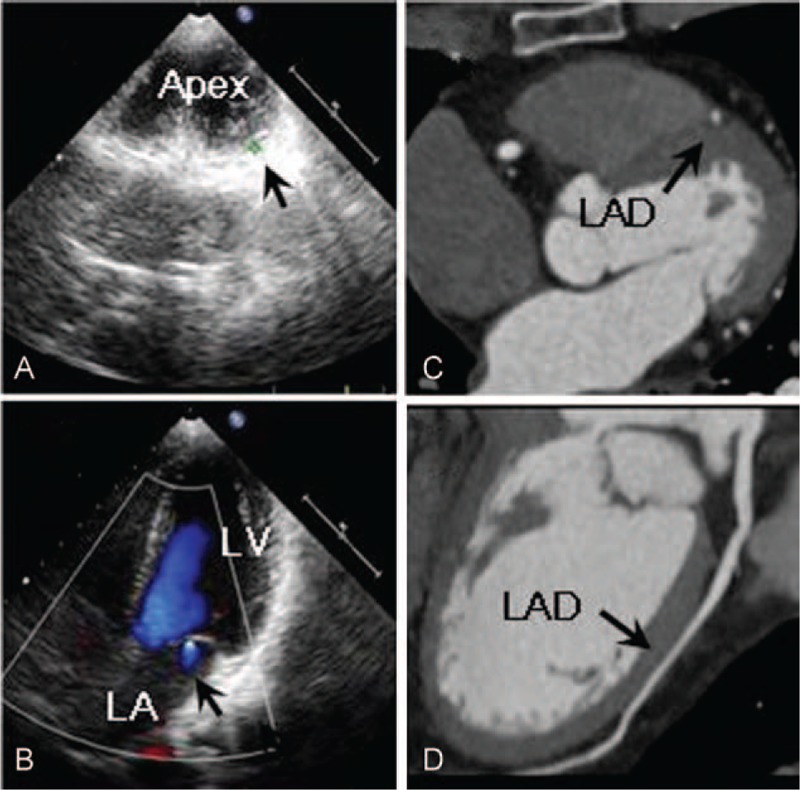
Images of echocardiograph (A–B) and cardiac CT angiography (C–D) of the patient who presents myocardial noncompaction with myocardial bridge. (A) Deep intertrabeculations recesses in the endocardial wall of left ventricular particularly in apex free wall; (B) mitral valve with regional regurgitation; (C) left descending artery (LAD) is surrounding by cardiac muscles; and (D) LAD keeps its shape against the cardiac muscles, leaving no space between the segment and cardiac muscle.

Electrocardiograph and chest x-ray showed normal. Echocardiograph revealed increased trabeculations below the level of papillary muscle of left ventricle (LV), and deep intertrabecular recesses in the endocardial wall of LV particularly in apex free wall (Figure 1A) where the trabecular thickness was 9 mm and the cardiac muscles became thinner. In addition, the apical impulse was decreased. Regional mitral regurgitation (Figure 1B) with normal movement was observed. The functional measurement (M-mode, Tiech method) showed LV ejection fraction (EF) of 57%.

Coronary computerized tomography (CT) scan showed that right coronary artery backbone opened in right coronary sinus, the trunk development was good, the tube wall was smooth, the lumen unobstructed, and plaque formation was not observed. Posterior descending coronary artery came from right coronary artery. Left descending artery (LAD) was developing well, the segment (by 41.0 cm distance to the opening of it and closed to middle part of it) was surrounding by cardiac muscles (Figure 1C), keeping its shape against the cardiac muscles, leaving no space between the segment and cardiac muscle, and the length of segment was 38.9 cm (Figure 1D). Left main coronary artery opened in left coronary sinus, it and left circumflex were developed well, the tubes wall was smooth, the lumens unobstructed, and plaque formation was not observed.

The diagnosis of the patient was NVM, coexisting with MB of LAD. Therapeutics interventions included: lifestyle cares: avoid strenuous exercise, settle good life habits, keep enough resting or sleeping time; taking Trimetazidine: 60 mg/time, 3 times daily, anti-ischemic agent, improves myocardial glucose utilization through inhibition of fatty acid metabolism; taking Shensongyangxin capsule: 1.2 g/time, 3 times daily, a traditional Chinese medicine, containing ginseng, ophiopogon japonicus, schisandra, and so on, improves myocardial cell metabolism, ischemia, and arrhythmia; and taking compound tablet of red sage root: a traditional Chinese medicine. More than 3 medications were taken discontinuously for 3 to 4 months per year. The patient is followed up until now there are no adverse and unanticipated events, his general condition is ok. EF is 77% from echocardiograph. Other parameters from echocardiograph and electrocardiograph are not changed. Coronary CT scan is not reperformed.

All in all, according to the patient's symptom (chest prickling-like pain) and mainly image examinations, the NVM was diagnosed by echocardiography, and MB was diagnosed by coronary CT scan. Diagnostic challenge is that doctor should know NVM can be developed with MB, and the complete diagnosis of NVM and MB should be made by different image studies.

The patient does not share his perspective with other parts. The patient gives informed consent to our work.

## DISCUSSION

Myocardial noncompaction is an abnormal ventricular muscle structure because of myocardial congenital aplasia. The pathogenesis of the NVM is not clear. Similar to other cardiac defects, it may be associated with the genetic factor (familial) or secondary causes (sporadic), on the basis of other congenital heart diseases, such as coronary artery congenital malformations, which cause ventricular pressure overload and myocardial ischemia, resulting prevention of the normal embryonic myocardium gradual compaction from interwoven myocardial fibers like “spongy” meshwork. Coronary circulation is established in general at the same time when above the process of ventricular myocardium compaction, during 5 to 8 weeks of human fetal life.^4^

Familial occurrence is frequent with single gene point mutations, for example, G4.5 and α-dystrobrevin.^5^ These different mutation genes were identified in sarcomere protein, resulting in specific myocardial morphological changes with developmental cardiopathies and functional changes with cardio-normal function disrupted. The prevalence rate of NVM is unknown.

Although MB is in general is not associated with family history, it is congenital in origin and likely reflects an evolutionary remnant in the genetic code.^6^ A study on the genetic mutations in myocardial bridging is helpful to research on MB accompanying with other congenital diseases. The prevalence rate of MB at autopsy is higher (5%–56%) than angiography (0.5%–12%),^6^ the reason for this is most likely the sensitivity of the different methods applied; small amounts of myocardial bridging often are undetectable with angiography, whereas 12% to 50% of the reported patients with NVM had family history.^7^ In the largest series of patients with NVM, the prevalence was 0.014% of patients referred to the echocardiography laboratory.^8^

Therefore, a link between the NVM and MB is coincidently embryogenesis. Currently, the diagnosis of NVM or MB is frequently overlooked, misdiagnosed, or delayed because of ignored finding or lack of the knowledge of the diseases. Meanwhile, it was discovered more chances than before as increasing the knowledge and new technologies developed. From aspect of embryonic development described above, NVM may occur with the process of development of NVM.^4^

Currently, to diagnose the NVM using echocardiograph refers to 2 sets of criteria in general: one is Jenni criteria^9^ and another is Chin criteria.^10^ The former is used wider that the typical 2 layers of different myocardial structure (inner loose vs outer tight ≧2) can be seen in the apex (>80%), lateral wall or inferior wall of heart. The latter method of analysis is complex and fails to clinical application; it measures the height between the recess and the trabecula. It is important that in both sets of criteria, there are no other abnormalities of cardiac structure, such as coronary artery anomalies or semilunar valve obstruction.

The case we report here was male, 34 years, had chest pain and relatively low blood pressure, had no family history of heart disease, ever and easily misdiagnosed as low blood pressure and nervous breakdown. The patient was finally diagnosed as NVM accompanied with MB. His NVM was detected by echocardiography, which is the diagnostic modality of choice for NVM commonly. In general, the excessively prominent trabecular meshwork was observed at the left ventricular apex most; near the mitral valve site least, which were consistent with the finding from current patient. The patient's MB was detected by noninvasive coronary CT scan.

So far there were only 3 case reports of MBs presenting with left ventricular myocardial noncompaction,^11–13^ all of them were from Turkey. This report was first case of such disease from China. Taking the 4 cases together indicates that such disease may appear in different races in different countries.

The strength of current case is that it is unique and interesting. Different from other 3 reported cases,^13,14^ the current case was from China rather than from Turky, had normal electrocardiograph appearances rather than abnormal morphology, and had mitral regurgitation with low EF rather than only low EF or left ventricular hypertrophy except for NVM on echocardiography. Similar to 3 reported cases, the patients were all male.

Characteristics of NVM coexisting with MB were shown in Table 1 according to other 3 case reports^13,14^ and current case. The findings from these patients (Table 1) indicate that MB coexisting with NVM is generally male, young, with low blood pressure, chest pain, ECG abnormal, MB involving LAD, and NVM involving LV. More patients are needed for further studies.

**TABLE 1:**
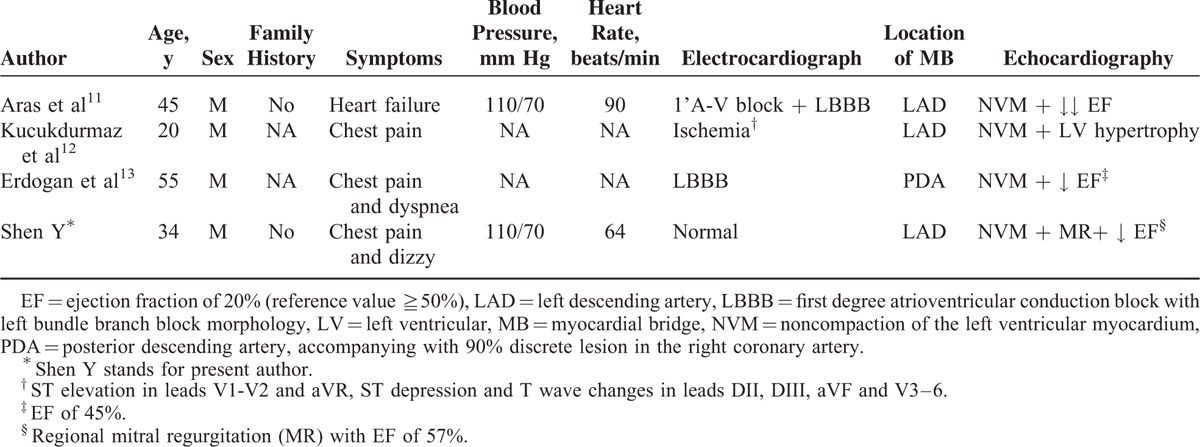
Characteristics of NVM Coexisting With MB

The limitations in this case was that we did not observe the systolic compression (“milking effect”) of the coronary artery because the patient declined to be performed cardiac CT angiography, which is the current gold standard for diagnosing MBs, although in patients with thin bridges, the milking effect may be missed. Myocardial bridging may only be identifiable after percutaneous transluminal coronary angioplasty when higher intravascular pressures and reversed hypokinesis unmask myocardial bridging.

Except for above cases of MB presenting with NVM, another article was related and interesting that NVM accompanying with an abnormal origin of a single coronary artery.^14^ The patient was a 75-year-old woman; had resting dyspnea for several days. Two-dimensional echocardiography revealed global impairment of systolic function with an enlarged LV; and multiple recesses with myocardial trabeculations at the apex and mid-part of the LV. Dynamic contrast magnetic resonance imaging showed that a coronary artery originated from the proximal ascending aorta. The conclusion of this case is similar to above 3 cases that NVM can present with other life-threatening cardiovascular deformities and different imaging studies are necessary for a comprehensive diagnosis.

In conclusion, both of the myocardial noncompaction and MB are congenital defects. NVM is spongiform cardiomyopathy, easily results in heart failure. MB is considered a risk factor of atherosclerosis. We report current patient indicating that NVM can be developed with MB. The complete diagnosis of NVM and MB should be made by different image studies. The diagnosis of NM can be made by 2-dimensional and color Doppler echocardiography; the diagnosis of MB can be made by coronary CT scan and/or better cardiac CT angiography.
